# Vaginal Hysterectomy for Cancer

**Published:** 1885-08

**Authors:** 


### Vaginal Hysterectomy for Cancer.

At the recent meeting of the American Medical Association, Dr. A. Reeves Jackson, of Chicago, read on this subject, basing his conclusions upon the results in 256 cases as compiled by Dr. Paul F. Mundé, and in 201 cases as reported by Schroeder and Olshausen. A mortality of 24.6 per cent. in the 256 by Mundé, he claims, is too small, as the cases were "picked," some of the unsuccessful being suppressed. The mortality reported by Schroeder and Olshausen is 38.6 per cent. His conclusions are:

(1) Any operation for cancer, which does not completely remove the disease, will be followed by recurrence.

(2) During life, the diagnosis of the extent of cancerous disease originating in any part of the uterus is at present impossible; hence, no operative procedure can afford a guarantee of complete removal.

(3) In view of this necessary doubt, no operation is justifiable which greatly endangers life, provided other and safer methods of treatment are available.

(4) Vaginal hysterectomy has sacrificed the lives of more than one-third of those who have been subjected to it; the mortality of the operation, when done by those of greatest skill and experience, being over 36 per cent.

(5) Other methods of treatment, attended by not more than one-sixth to one-fourth the mortality of vaginal extirpation, are equally efficient in ameliorating the symptoms and retarding the progress of the disease, and these have been followed by as good, or better, ultimate results; hence, they should be preferred.

(6) Hysterectomy does not avert or lessen suffering; it destroys, and does not save life. It is, therefore, not a useful, but an injurious operation, and, being such, it is unjustifiable, and ought to be abandoned.

As opposed to these objections of Dr. Jackson, Dr. Mundê contends that there is a field of usefulness for vaginal hysterectomy for the following reasons: The percentage of mortality is constantly decreasing, as the experience of operators increases, and as they select cases favorable for operation, as proven by the recent report of twenty-four operations by Feitch, with only two deaths. In estimating the percentage of recurrence, at Mundê's time of writing, only eighty-two cases were available, two years having elapsed since operation. Of these, 39.2 per cent. remained free from recurrence; while, from removal of the cervix alone, the percentage is only 25, and from various other methods it is only 21.8 per cent.

These eighty-two are among the earlier cases operated upon; hence, the percentage of recurrence would naturally be greater than in those better selected. In those cases where there is recurrence after hysterectomy, the amount of suffering is much less than before the operation, and without the same hemorrhages, as attested by the observations of many operators.

